# Characteristics of drugs safety signals that predict safety related product information update

**DOI:** 10.1002/pds.4446

**Published:** 2018-05-24

**Authors:** Widya N. Insani, Alexandra C. Pacurariu, Aukje K. Mantel‐Teeuwisse, Liana Gross‐Martirosyan

**Affiliations:** ^1^ Division of Pharmacoepidemiology and Clinical Pharmacology, Utrecht Institute for Pharmaceutical Sciences (UIPS) Utrecht University Utrecht The Netherlands; ^2^ Dutch Medicines Evaluation Board Utrecht The Netherlands; ^3^ Department of Medical Informatics Erasmus University Medical Center Rotterdam The Netherlands

**Keywords:** drug labeling, pharmacoepidemiology, pharmacovigilance, post‐market drug safety, safety signal

## Abstract

**Purpose:**

Investigation of drug safety signals is one of the major tasks in pharmacovigilance. Among many potential signals identified, only a few reflect adverse drug reactions requiring regulatory actions, such as product information (PI) update. Limited information is available regarding the signal characteristics that might predict PI update following signal evaluation. The objective of this study was to identify signal characteristics associated with PI updates following signal evaluation by the European Medicines Agency Pharmacovigilance Risk Assessment Committee during 2012 to 2016.

**Methods:**

A comparative study was performed based on data from 172 safety signals. Characteristics of signals were extracted from the European Pharmacovigilance Issues Tracking Tool database. Multivariable logistic regression analysis was used to assess the relationship between signal characteristics and the decision to update the PI.

**Results:**

Multivariable logistic regression analysis showed that the presence of evidence in multiple types of data sources (adjusted odds ratio [OR] 7.8 95% CI [1.5, 40.1]); mechanistic plausibility of the drug‐event association (adjusted OR 3.9 95% CI [1.9, 8.0]); seriousness of the event (adjusted OR 4.2 95% CI [1.3, 13.9]); and age of drugs ≤5 years (adjusted OR 3.9 95% CI [1.2, 12.7]) were associated with the decision to change the PI (*P* < 0.05).

**Conclusions:**

This study identified 4 characteristics of drug safety signals that have shown to be associated with PI changes as outcome of signal evaluation. These characteristics may be used as criteria for selection and prioritization of potential signals that are more likely to necessitate product information updates.

**KEY POINTS**

- Studies investigating drugs safety signals characteristics that might predict safety‐related product information changes are lacking.
- Confirmation of the signals in multiple types of data sources, the presence of mechanistic plausibility of a drug‐event association, seriousness of the events, and age of drugs ≤5 years were associated with the decision to update the product information.
- These criteria may be used for the selection or prioritization of the signals that are more likely to provide new safety information.

## 1 INTRODUCTION

During the development of medicinal products, identification of adverse reactions, particularly rare adverse reactions and those with long latency, is limited. Pre‐approval trials are typically conducted with relatively small number of patients in a limited length of time. Selective enrollment of participants may also limit the generalizability in the postmarketing environment. Continuous safety surveillance is thus essential to ensure patient safety.^1^, ^2^, ^3^

A safety signal is defined as the information suggesting a new potential association or new aspects of a known association between medicines and adverse event(s) that warrant further investigation.^4^ Signals can be generated from a wide range of sources, eg, a review of spontaneous case reports, data from active surveillance system, or from literature findings. To assess whether the signals represent true risks associated with medicines, several steps of additional data collection and analysis should be conducted. Based on such assessment, appropriate actions should be decided upon, eg, regulatory actions, such as amendment of product information (PI), initiation of referral, urgent safety restrictions; additional data needed, such as post‐authorization safety studies; and no actions needed beside routine pharmacovigilance.^5^

In the European Union, the decision‐making process related to safety signals is coordinated by the European Medicines Agency (EMA) Pharmacovigilance Risk Assessment Committee (PRAC).^6^ The PRAC is responsible for recommendations following signal assessment. During the first 18 months since its operation, 59% of the signals discussed at the PRAC resulted in regulatory actions, mostly updates of safety‐related information in the PI.^7^ Such amendments can include the addition of adverse drug reaction (ADR) or new aspect of current ADR, contraindication, warning and precaution related to the drug safety, etc.^8^

Several signal characteristics have been postulated to help in signal assessment, including strength of evidence, public health impact, and the novelty of drugs and/or safety issues.^9^, ^10^, ^11^, ^12^ However, information on the predictive validity of these criteria, ie, whether they can predict if the safety signal reviewed requires a PI update is lacking. Therefore, we conducted this study to identify signal characteristics associated with the decision to update the PI following the signal assessment.

## 2 METHODS

The list of safety signals discussed at the PRAC since September 2012 until May 2016 was obtained from the publicly available data on the website of the EMA.^13^ We included signals which resulted in PI updates and those which were closed with no PI update or other regulatory actions. The signals with no assessment conclusion available at the time of data collection was excluded because the assessment was still ongoing. Signals that were further assessed in other regulatory procedures and those which resulted in regulatory actions other that PI updates were excluded. Each signal comprised information on the adverse event and the suspected drug. The adverse events were classified using the Medical Dictionary for Regulatory Affairs (MedDRA) 19.1 based on the System Organ Classification (SOC) code.^14^ The suspected drugs were categorized based on the Anatomical Therapeutic Chemical (ATC) classification codes by the World Health Organization (WHO).^15^ Signals characteristics were extracted from the European Pharmacovigilance Issues Tracking Tool (EPITT) database. EPITT is a web‐based system facilitating the tracking and sharing of safety information on medicinal products for human use established by the EMA.^16^

### 2.1 Signals characteristics

Characteristics that are potentially important during signal assessment were pre‐defined and classified in 3 categories, namely the strength of evidence, public health impact, and the novelty of the drug. The rationale and definitions used for categorization of the signals are provided below.

#### 2.1.1 Characteristics related to the strength of evidence

- Source of evidence

The presence of signals in a wide range of additional sources may strengthen the evidence supportive of the signals.^17^, ^18^ For each signal, we extracted the type of data source providing supporting evidence for possible causal association between the drug and the event, ie, spontaneous case reports, observational studies, clinical studies, and pre‐clinical studies.

- Mechanistic plausibility

The presence of mechanistic plausibility is an important factor supporting the association.^18^ Mechanistic plausibility was considered available when either a hypothesized or established mechanism was discussed during signal evaluation.

- Presence of disproportionate reporting

Increased frequencies of the case reports concerning a specific drug‐event association in comparison with general reporting frequencies may indicate a new potential signal.^17^, ^19^, ^20^ The signals were considered disproportionate if (1) the lower bound of the 95% confidence interval of proportional reporting ratio (PRR) was equal or greater than one.^5^ (2) The value of Empirical Bayes Geometric Mean (EBGM) was equal or greater than 2.5.^21^

- Positive dechallenge or rechallenge

The presence of positive dechallenge and rechallenge might be important in establishing causality based on individual narratives of the reported cases. 20, 22 Positive dechallenge was considered present if there was at least 1 spontaneous post‐marketing report where the adverse event disappeared after the concerned drug was withdrawn. Positive rechallenge was noted as present if the assessment of a signal included at least 1 report where the adverse event reappeared after restarting the drug.

- Possible class effect

Knowledge that drugs from the same pharmacological class produce the same adverse effect might strengthen the evidence for a signal. The signals were classified as reflecting possible class effects if during signal assessment it was mentioned that the suspected event is labeled for other drugs from the same class.^22^

### 2.2 Criteria related to the public health impact

#### 2.2.1 Seriousness of the events

Serious events usually have an increased public health importance compared with non‐serious ones.^18^, ^23^ The events were classified as serious if they were included in the EMA's important medical events list. This list includes medical events that are fatal, life‐threatening, require hospitalization or prolong existing hospitalization, result in significant disability, or cause congenital anomaly/birth defect.^24^

#### 2.2.2 Criteria related to drug novelty

New risks are more likely to be observed in newer drugs.^9^, ^12^ The age of a drug was calculated from the date of the first authorization until the date when the PRAC recommendation was made.^25^ The drugs were grouped in the following age categories: 0 to 5; 5 to 10; 10 to 15; and ≥15 years.

## 3 DATA ANALYSIS

Descriptive analysis was used to compare characteristics of signals between the groups which resulted in safety‐related PI changes and without. To assess the influence of various characteristics on the PI update, first, a univariate logistic regression analysis was performed. Criteria that were associated with PI changes with a *P*‐value<0.1 in the first analysis were then included in a multivariate logistic regression model. Subgroup analysis was performed to investigate whether different signal characteristics were associated with the updates of section 4.8 (undesirable effects) as compared with updates of section 4.4 (special warnings and precautions for use) of the PI. The results were expressed as odds ratios (ORs) with 95% confidence intervals (CIs). *P* < 0.05 defined statistical significance in the main analysis, while for subgroup analysis, statistical significance was set at *P* < 0.1. Analysis was performed using Stata 11.2.

## 4 RESULTS

**Figure 1:**
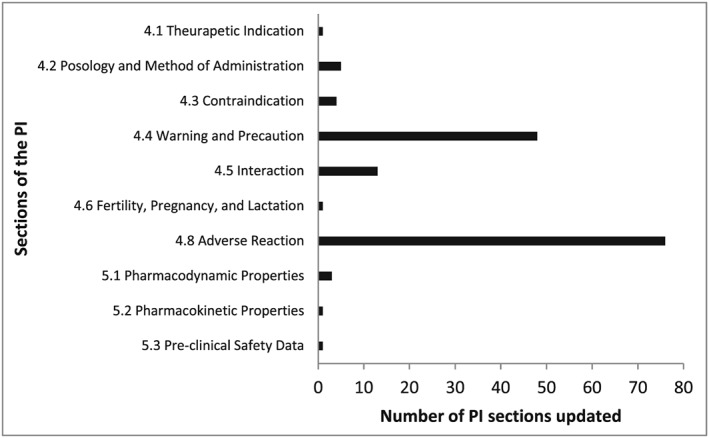
Section of product information updated following safety signal evaluation by the PRAC during September 2012 to May 2016

During the study period (September 2012–May 2016), 300 signals were assessed at the PRAC. After excluding non‐eligible signals, ie, signals which were further investigated in other regulatory procedures (*n* = 94), signals which resulted in regulatory actions other than PI updates (*n* = 20), signals assessments were still ongoing (*n* = 4), and signals for which a full assessment could not be retrieved (*n* = 10), 172 signals remained for the analysis. The most frequently identified ADR were related to skin and subcutaneous tissue disorders (10%). Most frequently involved drugs were antineoplastic and immunomodulating drugs (31%) (Table 1). A total of 101 signal assessments resulted in PI updates, and 71 were assessed and closed with no actions beside routine pharmacovigilance. Most PI updates involved the revision of the ADR section (section 4.8), followed by the warnings and precautions section (section 4.4). Updates resulted from a signal could involve the revision of more than 1 PI sections (Figure 1).

**Table 1:** Adverse event and suspected drug of included signals

	*n* (%)
Adverse events (SOC)	
Skin and subcutaneous tissue disorders	18 (11)
General disorders and administration site condition	15 (9)
Blood and lymphatic system disorders	12 (7)
Nervous system disorders	12 (7)
Metabolism and nutrition disorders	11 (6)
Cardiac disorders	11 (6)
Gastrointestinal disorders	10 (6)
Others (less than 5%)	83 (48)
Drugs class	
Antineoplastic and immunomodulating agents	53 (31)
Nervous system	30 (17)
Antiinfective for systemic use	22 (13)
Alimentary tract and metabolism	14 (8)
Cardiovascular system	13 (8)
Blood and blood forming organs	11 (6)
Others (less than 5%)	29 (17)

Spontaneous case reports were the most common source of evidence, supporting 87% of the signals. We also found that 43% of the signals which resulted in PI updates were assessed based on evidence coming from spontaneous case reports only. In the univariate logistic regression model, the presence of evidence from spontaneous case reports was associated with PI update (unadjusted OR 2.2 [95% CI 0.9, 5.6]). Greater magnitude of difference was seen in the signals supported by ≥3 types of sources (unadjusted OR 7.4 [95%CI 1.6, 33.3]). The presence of biological plausibility of the drug‐event association led to more PI updates (unadjusted OR 4.2 [95% CI 2.2, 8.0]).

Although the proportion of positive dechallenge and rechallenge results was higher among the signals resulted in PI updates, the differences were not statistically significant. The proportion of serious events was significantly higher among the signals resulted in PI update (unadjusted OR 2.9 [95% CI 1.0, 8.2]). Signals concerning younger products, ie, ≤ 5 years old, resulted more often in PI updates (unadjusted OR 2.4 [95% CI 0.8, 7.1]) (Table 2).

**Table 2:** Univariate analysis comparing characteristics of signals with and without PI update

Characteristics	Signals Resulted in PI Update (*n* = 101 (%))	Signals were Closed with no Actions at the Time (*n* = 71 (%))	Crude Odds Ratio (95% CI)	*P*‐Value
Strength of evidence				
Source of evidence				
Case reports	92 (61)	58 (39)	2.2 (0.9, 5.6)	**0.07**
Observational studies	24 (56)	19 (44)	0.8 (0.4, 1.7)	0.65
Clinical studies	38 (62)	23 (38)	1.2 (0.6, 2.3)	0.48
Pre‐clinical studies	18 (72)	7 (28)	1.9 (0.7, 5.0)	0.15
≥ 2 sources	50 (60)	33 (40)	1.1 (0.6, 2.0)	0.69
≥ 3 sources	18 (90)	2 (10)	7.4 (1.6, 33.3)	**0.00**
Mechanistic plausibility	74 (73)	28 (27)	4.2 (2.2, 8.0)	**0.00**
Disproportionate reporting	40 (56)	31 (44)	0.8 (0.4, 1.5)	0.59
Presence of dechallenge/rechallenge results				
Positive dechallenge	36 (61)	23 (39)	1.1 (0.6, 2.1)	0.65
Positive rechallenge	34 (65)	18 (35)	1.4 (0.2, 7.6)	0.24
Possibility of a class effect	14 (67)	7 (33)	1.4 (0.5, 3.8)	0.43
Public health impact				
Seriousness of the event	95 (61)	60 (39)	2.9 (1.0, 8.2)	**0.04**
Novelty				
Age of drugs				
0–5 years old	16 (76)	5 (24)	2.4 (0.8, 7.1)	**0.09**
6–10 years old	12 (44)	15 (56)	0.5 (0.2, 1.1)	0.10
10–15 years old	19 (59)	13 (41)	1.0 (0.4, 2.2)	0.93
>15 years old	54 (59)	38 (41)	0.9 (0.5, 1.8)	0.99

The bold data are the criteria with *P*‐ value < 0.1 in univariate analysis, which were then included in multivariate logistic regression model

In the multivariate logistic regression model, 4 signals characteristics were shown to be independently associated with PI update, ie, the presence of evidence in ≥3 types of sources (adjusted OR 7.8 [95% CI 1.5, 40.1]), the mechanistic plausibility of drug‐event association (adjusted OR 3.9 [95% CI 1.9, 8.0]), seriousness of the events (adjusted OR 4.2 [95% CI 1.3, 13.9]), and age of drugs ≤5 years (adjusted OR 3.9 [95% CI 1.2, 12.7]) (Table 3).

**Table 3:** Multivariate logistic regression model showing the predictors of PI update

Characteristics	Odds Ratio (95% CI)	*P*‐Value
Signals supported by case reports	1.6 (0.5, 4.4)	0.34
Signals supported by ≥3 types of sources	7.8 (1.5, 40.1)	**0.01**
Mechanistic plausibility	3.9 (1.9, 8.0)	**0.00**
Seriousness of the event	4.2 (1.3, 13.9)	**0.01**
Age of drugs ≤5 years	3.9 (1.2, 12.7)	**0.02**

The bold data are the criteria with P value <0.1 in a multivariate logistic regression model

Based on the subgroup analysis where signals resulting in update of section 4.4 were compared with signals resulting in update of section 4.8, the seriousness of the event, disproportional reporting, and age of the drug being 0 to 5 years old were associated with an update of section 4.4, but not section 4.8. On a contrary, the availability of a positive dechallenge and rechallenge, the possibility of a class effect, and presence of evidence in ≥3 types of sources were associated with an update of section 4.8 but not section 4.4. The drugs in older age category (6–10 years) were less likely to be associated with any PI updates (Table 4).

**Table 4:** Subgroup analysis comparing characteristics of signals resulting in update of section 4.4, 4.8, and both sections

Characteristics	Crude Odds Ratio (95% CI), *P*‐Value		
	Signals Resulted in Update of Section 4.4 (*n* = 13)	Signals Resulted in Update of Section 4.8 (*n* = 40)	Signals Resulted in Update of Both Sections (*n* = 37)
Strength of evidence			
Source of evidence			
Case reports	1.3 (0.2, 6,7), *P* = 0.71	**3.1 (0.8, 11.2), *P* = 0.09**	**8.8 (1.1, 70.1), *P* = 0.04**
Observational studies	1.2 (0.3, 4.4), *P* = 0.76	0.6 (0.2, 1.7), *P* = 0.42	1.0 (0.4, 2.4), *P* = 0.97
Clinical studies	1.3 (0.3, 4.4), *P* = 0.67	1.2 (0.5, 2.8), *P* = 0.58	1.4 (0.6, 3.2), *P* = 0.40
Pre‐clinical studies	0.7 (0.1, 6.7), *P* = 0.80	2.2 (0.7, 6.8), *P* = 0.14	2.5 (0.8, 7.6), *P* = 0.10
≥ 2 sources	1.3 (0.4, 4.3), *P* = 0.62	1.0 (0.4, 2.2), *P* = 0.91	1.5 (0.6, 3.3), *P* = 0.31
≥ 3 sources	2.8 (0.2, 34.2), *P* = 0.40	**10.0 (2.20, 49.1), *P* = 0.00**	**9.5 (1.9, 47.5), *P* = 0.00**
Mechanistic plausibility	**8.4 (1.7, 41.0), *P* = 0.00**	**2.5 (1.1, 5.6), *P* = 0.02**	**4.7 (1.9, 11.6), *P* = 0.00**
Disproportionate reporting	**0.2 (0.04, 1.1), *P* = 0.07**	1.1 (0.4, 2.3), *P* = 0.89	1.1 (0.4, 2.4), *P* = 0.82
Presence of de−/rechallenge results			
Positive dechallenge	0.3 (0.1, 1.8), *P* = 0.23	**2.0 (0.9, 4.6), *P* = 0.07**	0.8 (0.3, 2.1), *P* = 0.77
Positive rechallenge	0.2 (0.02, 2.0), *P* = 0.19	**3.2 (1.4, 7.3), *P* = 0.00**	1.4 (0.5, 3.3), *P* = 0.43
Possibility of a class effect	1.6 (0.3, 9.0), *P* = 0.55	**2.6 (0.9, 7.7), *P* = 0.07**	0.5 (0.1, 2.6), *P* = 0.43
Public health impact			
Seriousness of the event	**4.3 (1.1,16), *P* = 0.03**	2.2 (0.8, 6.2), *P* = 0.10	2.2 (0.8, 6.1), *P* = 0.12
Novelty			
Age of drugs			
0–5 years old	**3.96 (0.8, 19.1), *P* = 0.08**	1.1 (0.2, 4.7), *P* = 0.92	2.0 (0.5, 7.6), *P* = 0.27
6–10 years old	0.3 (0.03, 2.5), *P* = 0.28	0.9 (0.3, 2.4), *P* = 0.88	**0.2 (0.04, 0.9), *P* = 0.04**
10–15 years old	1.3 (0.3, 5.5), *P* = 0.68	1.1 (0.4, 2.9), *P* = 0.82	0.8 (0.2, 2.4), *P* = 0.78
>15 years old	0.7 (0.2, 2.4), *P* = 0.62	0.8 (0.3, 1.8), *P* = 0.72	1.2 (0.5, 2.8), *P* = 0.55

The bold data are the criteria with *P*‐value < 0.1 which were considered influential in subgroup analysis

## 5 DISCUSSION

In this study, we examined several drug safety signals characteristics that might predict ADR requiring PI update. We found that the presence of evidence in multiple types of data sources, mechanistic plausibility of event‐drug association, seriousness of the event, and age of drugs ≤5 years old were associated with the decision to update the PI.

Spontaneous case reports remain an important source of ADR detection. Spontaneous reports often contain essential information necessary for causality assessment not available in other sources of data, such as plausible time course for development of ADR following initiation of the drug and information on dechallenge and rechallenge.^26^ However, we found that there was no distinct type of data source that was independently associated with signals requiring PI updates. It is rather the replication of the signal in multiple additional data sources that might indicate stronger evidence, as confirmed in our study. This finding is comparable with previous studies showing that multiple evidence sources supported the decision to conduct regulatory actions, eg, Food and Drug Administration safety labeling changes and drugs withdrawals in France.^27^, ^28^ Multiple evidence sources was also one of the criteria employed in the safety signals prioritization framework developed by several institutions.^11^, ^29^

Another criterion significantly associated with a PI update is the presence of a mechanistic plausibility of the drug‐event association, either an established or a hypothesized mechanism. Some true safety signals did not include a confirmed mechanism, but the occurrence of adverse events was mechanistically plausible, providing additional evidence supportive of the association. In addition, we also found that in several signal assessments, unlikeliness of a mechanistic plausibility constituted one of the arguments to reject the signals, eg, in the signal of glioblastoma and other brain neoplasms related to adalimumab and infliximab. Due to the size of their molecules, it was considered unlikely that these products would cross the blood brain barrier and caused malignancies in the brain.^30^

Seriousness of the event was another independent predictor of PI update in our analysis. Many serious events addressed by the PRAC in the recent years concerned events included on the Designated Medical Event term list, which are by definition serious events that are in general, more likely to be caused by drugs.^31^ Therefore, the selection of signals discussed in the PRAC might have been initially skewed towards more serious events, as confirmed by the fact that majority of the signals included in the analysis were serious events. Our finding was comparable with previous study by Puijenbroek et al showing that seriousness of the adverse event was a determinant during signals selection process.^9^ Serious reports might possess greater public health importance, providing the signals the precedence to be prioritized for evaluation.

In the subset analysis, the seriousness of the event was associated with the update of section 4.4, but not section 4.8. This implies that serious events may be more likely to prompt inclusion of a warning to inform health care professionals about a serious and potentially life‐threatening event; however, if there is sufficient evidence of causal association between an event and a drug, the adverse event will be included in the section 4.8 (undesirable effects) regardless of the seriousness.

We found that signals concerning newer products (≤ 5 years old) resulted more often in PI updates. At the time of drugs approval, only partial safety information was obtained due to several known limitations in pre‐marketing clinical studies.^1^, ^2^, ^3^ The rapid use of drugs by larger and more diverse population might contribute to the detection of new ADR during the first years after drugs approval. In addition, younger drugs are more intensively monitored by the regulatory authorities, eg, through a more frequent cycle for PSUR, which may also contribute to the detection of new safety issues. Besides, it has been shown by Weber that adverse reaction reporting peaks at the end of second year after authorization*,* ie, the so‐called Weber effect theory.^32^ Another study performed by McAdams et al also showed that during the first year after approval, the highest reporting of adverse event trend was observed.^33^ The subset analysis showed that the signals concerning very new products (≤5 years old) were associated with update of section 4.4, while the signals for somewhat older products (6‐10 years old) are less likely to be associated with any PI updates. On the other hand, we also found that more than half (59%) of the signals that resulted in PI updates concerned products that have been in the market for more than 15 years. PI updates in these cases were probably due to accumulation of evidence from different sources over the time. This finding highlights again the importance of continued pharmacovigilance for more mature products.^7^

Although positive dechallenge and rechallenge observed in individual spontaneous reports are important factors in establishing causality,^20^, ^22^ their presence was not a decisive factor for PI update in general. In the subset analysis, however, both positive dechallenge and rechallenge were associated with update of section 4.8 (as opposed to update of section 4.4). This could be explained by the fact that clinically meaningful dechallenge and rechallenge, especially when combined with other aspects of the narrative, eg*,* information regarding medical history of the patient, time to onset, the use of concomitant drugs, etc, are strongly suggestive of causal association and therefore signals with reported positive dechallenge and rechallenge are more likely to result in update of section 4.8.^34^, ^35^ The presence of positive dechallenge and rechallenge was however not associated with update of section 4.4, probably because a well‐established causal association is neither sufficient nor necessary for inclusion of a warning for health care providers in section 4.4 of PI. For example, common, non‐serious adverse events with well‐established causality may not require a special warning, while life‐threatening adverse events requiring a prompt action on behalf of prescribers to prevent irreversible harm in patients may warrant inclusion of a warning in section 4.4 even if available evidence is limited.

Our study showed that the presence of disproportionate reporting in safety databases was not necessarily associated with the decision to update the PI. Disproportionality analysis is subject to well‐known limitations, such as limited database quality inherent in voluntary reporting system, various confounding factors, and inability to provide actual denominator, ie, number of subjects who consumed drug of interest. These limitations might contribute to the occurrence of false signal of disproportionate reporting or alternatively true safety signals may appear without disproportional reporting.^34^, ^35^, ^36^, ^37^, ^38^ However, in the subset analysis, the presence of disproportionate reporting was associated with the update of section 4.4, implying serious events were probably often reported disproportionately.^39^

There have been a few attempts to combine criteria for signal prioritization in algorithms or frameworks to support the drug signal selection and prioritization process by different organizations responsible in managing drug safety issues.^10^ Enhancement of such decision support frameworks with valid predictive criteria may increase their accuracy. Our study provides a set of variables which show predictive value that can be incorporated in such frameworks to support the decision making when dealing with a large number of potential signals.

The strength of our study is that it is the first study investigating the predictive value of drug safety signals characteristics in terms of predicting whether the signals represent ADR requiring PI updates. In addition, we included signals investigated during the first 3.8 years since the establishment of the PRAC. Nevertheless, this study also had some limitations. We did not include signals addressed in other regulatory procedures, eg, the PSUR assessment procedure. However, because it was considered that the conduct of assessment was relatively similar, no significant change is expected. Furthermore, the performance of these characteristics is based on the signal assessment performed in the European Union. These criteria may perform differently in other databases comprising safety evaluation conducted in other geographic regions. Therefore, further studies are recommended if these variables are to be used in a different setting.

## 6 CONCLUSIONS

Our study highlighted that the presence of evidence in multiple type of sources, mechanistic plausibility, seriousness of the event, and age of drugs ≤5 years were the predictors of safety‐related PI changes. The characteristics related to the strength of evidence were particularly important for the update of section 4.8 (undesirable effects), while seriousness of the event was an important criteria for the changes in section 4.4 (warning and precaution for use). The knowledge related to these factors may be used to improve selection and prioritization of potential signals that are more likely to provide new safety information.

## ETHICS STATEMENT

The authors state that no ethical approval was needed.

## CONFLICT OF INTEREST

All authors have no conflicts of interests in this research. The views expressed in this article are the personal views of the author(s) and may not be understood or quoted as being made on behalf of or reflecting the position of the Dutch Medicines Evaluation Board or the European Medicines Agency or one of its committees or working parties.
